# SR Splicing Factors Promote Cancer via Multiple Regulatory Mechanisms

**DOI:** 10.3390/genes13091659

**Published:** 2022-09-16

**Authors:** Ledong Wan, Min Deng, Honghe Zhang

**Affiliations:** 1 Department of Pathology, Research Unit of Intelligence Classification of Tumor Pathology and Precision Therapy of Chinese Academy of Medical Sciences (2019RU042), Zhejiang University School of Medicine, Hangzhou 310058, China; 2 Cold Spring Harbor Laboratory, Cold Spring Harbor, NY 11724, USA; 3 Department of Pathology, First Peoples Hospital Fuyang, Hangzhou 311400, China

**Keywords:** SR proteins, RNA processing, cancer

## Abstract

Substantial emerging evidence supports that dysregulated RNA metabolism is associated with tumor initiation and development. Serine/Arginine-Rich proteins (SR) are a number of ultraconserved and structurally related proteins that contain a characteristic RS domain rich in arginine and serine residues. SR proteins perform a critical role in spliceosome assembling and conformational transformation, contributing to precise alternative RNA splicing. Moreover, SR proteins have been reported to participate in multiple other RNA-processing-related mechanisms than RNA splicing, such as genome stability, RNA export, and translation. The dysregulation of SR proteins has been reported to contribute to tumorigenesis through multiple mechanisms. Here we reviewed the different biological roles of SR proteins and strategies for functional rectification of SR proteins that may serve as potential therapeutic approaches for cancer.

## 1. Introduction

Advances in our understanding and therapy of cancer have been obtained from coordinated efforts to characterize genomic alterations in cancer through large-scale and ultra-deep sequencing and delicate models recapitulating clinical cancer features. However, unlike the heavily interrogated cancer genome, our understanding of RNA metabolism and translation is still in development. RNA-binding proteins (RBPs) recognize and assemble with RNA in RNA transcription. Different RBPs are recruited to or released from RNA following sequential events, such as 5′ capping, 3′ polyadenylation, RNA splicing, modification, intracellular trafficking, translation, and degradation. RBPs have various RNA-binding domains (RBD) that recognize specific RNA *cis*-elements or structures and auxiliary non-RNA binding domains with low amino acid complexity associated with protein–protein interaction [1,2,3]. RBPs can specifically interact with hundreds and thousands of transcripts and bind to sequence motifs and/or structures in RNA, thereby forming extensive regulatory networks and contributing to cell homeostasis [2].

**Figure 1:**
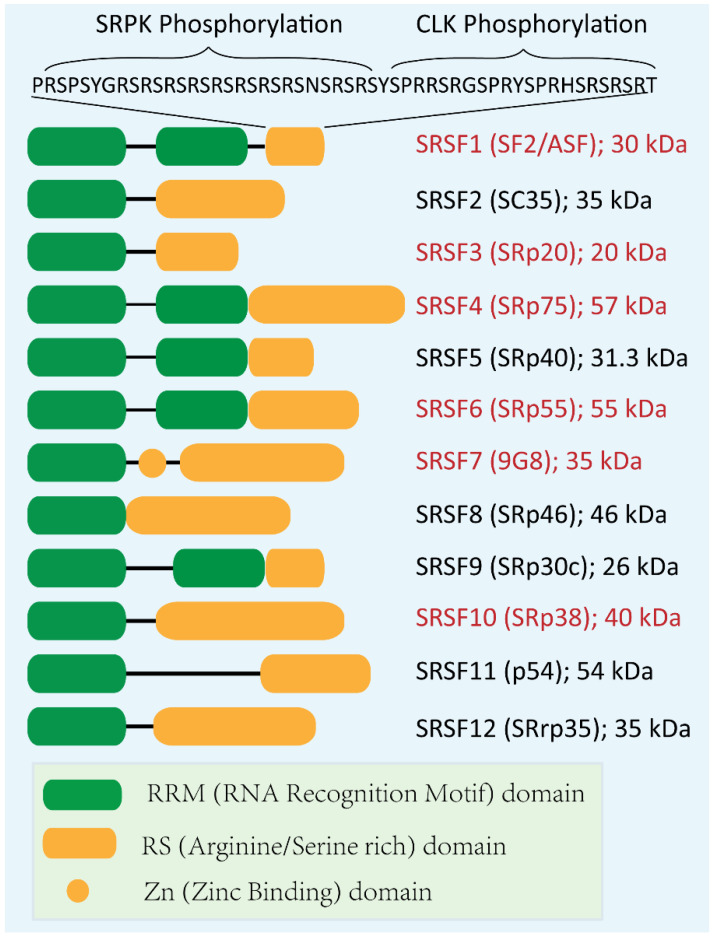
Schematic representation of the 12 human SR proteins. SR proteins are presented as SRSFs with aliases indicated in the parenthesis. Shuttling SR proteins (red letters) are reported to shuttle between nucleus and cytoplasm, whereas the others (black letters) have no shuttling activity. Phosphorylation of RS domain of SRSF1 by SRPK and CLK kinases is indicated as a representative at the top.

Serine/Arginine-Rich proteins (SR) belong to a family of RNA-binding proteins that consist of one or two RNA recognition motifs (RRMs) that specifically bind to RNA and a C-terminal RS domain enriched with arginine and serine residues involved in protein–protein interaction (Figure 1) [4,5]. SR proteins were first identified as potential nucleoproteins during transcription [6], and they were soon characterized as the splicing factors that mediate both constitutive and alternative splicing [7,8,9,10,11]. Additionally, several other SR protein functions have been reported, such as genomic stability, promoter selection, 3′ end processing, mRNA export, mRNA stability, and translation [12,13,14]. Here we review recent advances in our understanding of the SR proteins’ functions in RNA metabolism and translation and their roles in tumorigenesis. Targeting SR proteins and their downstream splicing changes should provide potential cancer therapeutic strategies.

## 2. Role of SR Proteins in Splicing

**Figure 2:**
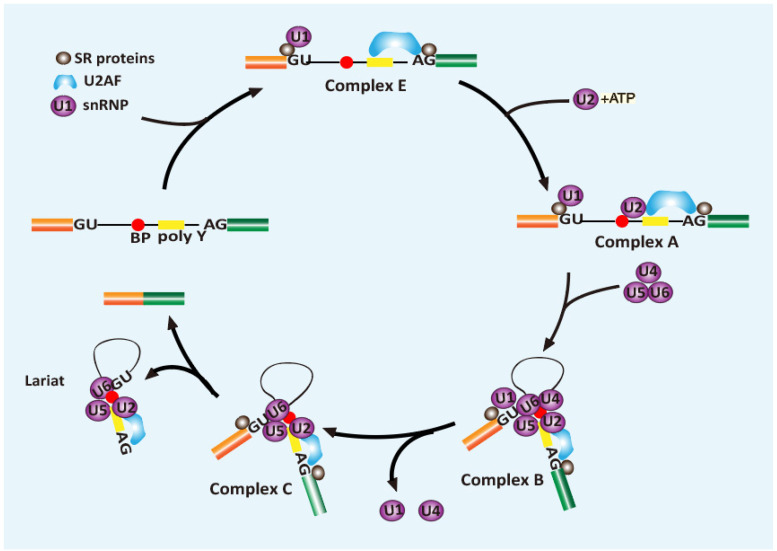
The role of SR proteins in spliceosome assembly. Many studies have identified SR proteins involved in nearly every step of the spliceosome assembly. Firstly, SR proteins could promote the formation of E complex and its binding to the 5’-splice site to facilitate base pairing between U1 snRNA with the splice site. Next, SR proteins recruit U2 snRNA to the branch point region of pre-mRNA to form complex A. Then SR proteins form a bridging complex across two splice sites via binding to U2AF at the 3′-splice site and U1 70K at the 5′-splice site, facilitating the recruitment of U4/U6 and U5 tri snRNP complex to form complex B.

RNA splicing is a critical process for the expression of >95% of human genes, during which the introns (non-coding sequences) dispersing throughout the primary transcripts are excised, and exons are ligated together [15,16]. RNA splicing involves two consecutive transesterification reactions that occur under the regulation of the spliceosome, a dynamic macromolecular machine that is comprised of proteins and small nuclear RNAs. The spliceosome contains over 300 components that were recruited and assembled onto the pre-mRNA, undergoing a series of dynamic changes via RNA–RNA, protein–protein, RNA–protein, and recently discovered protein–phosphoinositide interaction during the splicing reaction (Figure 2) [17,18]. The splicing reaction starts with U1 small nuclear ribonucleoprotein (snRNP) recognizing the 5′ splice site in an ATP-dependent manner; together with SF1, U2 auxiliary factors (U2AF1 and U2AF2) recruited to the branch point site (BPS), the polypyrimidine tract (PPT), and the 3′ splice site, respectively. U2 snRNP then binds to the BPS to displace and release SF1 in an ATP-dependent manner. This interaction is stabilized by the assembling of SF3a and SF3b protein complexes, as well as the U2AF that recognizes the 3’ splice site. Then the pre-assembled U4/U6/U5 tri-snRNP joins the pre-splicing complex to form a fully assembled spliceosome (complex B). The complex B further conformationally transforms into the catalytically active spliceosome by releasing U1 and U4. This active spliceosome cleaves the 5′ splice site and allows the ligation between the intron and branch point site, forming a lariat. Next, the spliceosome further transforms to bring the two exons close together and catalyzes the joining of the exons and the release of the lariat [19].

Though stoichiometric RNA and protein components are present in the spliceosome, some splice sites are generally not competitive to recruit a functional spliceosome. They need auxiliary elements nearby to facilitate regulating the splicing events, such as ESEs and ESSs (exonic splicing enhancers and silencers, respectively) in the exons, and ISEs and ISSs (intronic splicing enhancers and silencers, respectively) in the introns. As pivotal recognizers of these elements, SR proteins can bind to these elements and facilitate spliceosome assembling [11]. The function of SR proteins has been identified in nearly every step of the splicing procedure. Firstly, SR proteins generally recognize the ESE elements and promote the formation of E complex and its binding to the 5’-splice site by assisting U1 snRNA in recognizing the splicing site [11]. Next, SR proteins promote U2 snRNA interacting with pre-mRNA at the branch point region to form complex A. Then SR proteins, such as SRSF2, enforce the interaction between U2AF and U1 70K to bridge complexes across two splice sites, which further recruits U4/U6 and U5 tri snRNP complex to form complex B (Figure 2) [20]. Among these steps, the most critical function of SR proteins is documented in the early splice site recognition and initiation of spliceosome assembly. Through binding with a preferable sequence, SR proteins assist adjacent splice sites to compete in recruiting spliceosomes, which results in enhanced RNA splicing.

## 3. Role of SR Proteins in Transcriptional Elongation and Genomic Stability

**Figure 3:**
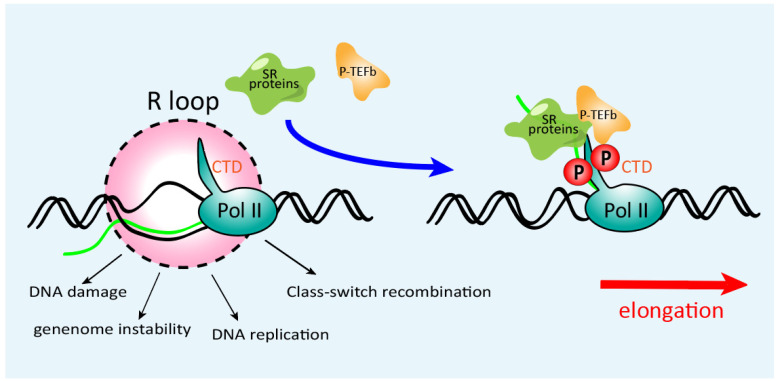
SR proteins in transcriptional elongation and genome stabilization. Through binding to nascent RNA, SR proteins hinder the R-loop formation and dynamically bridge P-TEFb to Pol II, which further catalyzes Pol II phosphorylation in its C-terminal repeat domain (CTD), promoting transcriptional elongation in many genes.

During transcriptional elongation, nascent RNA can displace the non-template strand and bind to template DNA to form an RNA:DNA hybrid (R-loop) that extends for about nine nucleotides [21]. R-loops have been extensively implicated in genome instability: the exposed non-template ssDNA becomes a vulnerable target to nucleases or other DNA modification enzymes [22]. Blockage or termination of transcription elongation gives rise to stabilized R-loops behind the stalled Pol II complex [22,23]. Transcription is processed functionally coupled with RNA transcript maturation, including 5′ capping, pre-mRNA splicing, cleavage/polyadenylation of nascent transcripts, and ribonucleoprotein assembly [24,25,26]. In addition to their functions in the spliceosome, SR protein family members also participate in the Pol II complex, contributing to transcriptional elongation (Figure 3) [27,28]. For example, SRSF2 can facilitate P-TEFb binding to Pol II, increasing Pol II phosphorylation at Ser2 positions in its C-terminal repeat domain (CTD) and promoting transcriptional elongation [29].

Additionally, SR proteins can attach to and prevent the pre-mRNA binding to template DNA. These features of SR proteins can suppress the R-loops formation, guarantee the correct transcriptional elongation, and thereby increase genome stability (Figure 3). SRSF1 knockout in chicken DT40 cells results in genome-wide DNA breaks and gross DNA recombination [12]. Moreover, knocking out SRSF2 in mouse embryo fibroblasts also results in catastrophic DBS, which may block the cell cycle at the S-phase checkpoint [30]. Overall, SR proteins hinder the formation of the R-loop by promoting transcriptional elongation and binding to nascent RNA to help release RNA from the template DNA when a splicing signal emerges, thereby safeguarding the genome stability.

## 4. Post-Splicing Activities of SR Proteins

**Figure 4:**
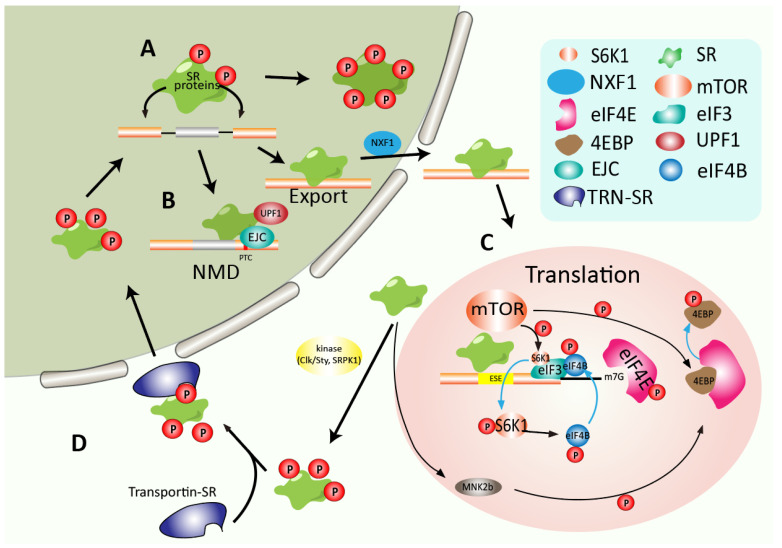
Regulation of SR proteins and their roles in mRNA export, translation, and decay. (**A**) SR proteins require phosphorylation to mediate spliceosome complex formation. With further dephosphorylation, SR proteins bind and export the mature mRNA into the cytoplasm. (**B**) SR proteins perform a significant role in NMD, which can alter NMD from taking place after mRNA export to the cytoplasm to coming up before mRNA release from the nucleus. (**C**) SR protein-bound mRNAs recruit the mTOR kinase resulting in the phosphorylation and release of 4E-BP, leading to enhanced translation initiation. SR proteins can enhance the mTOR kinase phosphorylating S6K1, which promotes translation initiation. Meanwhile, The SF2/ASF-dependent alternative splicing leads to Mnk2b isoform, which can activate translation. (**D**) Phosphorylated by kinase, such as SRPK1 or/and Clk, SR proteins are specifically recognized and transported into the nucleus by Transportin-SR protein.

In addition to their activities in the nucleus, the nucleo-cytoplasmic shuttling property of SR proteins confers their noncanonical function in mRNA export, translation, and decay (Figure 1 and Figure 4) [11,31].

### 4.1. SR Proteins Promote Mature RNA Export from the Nucleus

After processing, mRNA is exported to the cytoplasm, where the translation is highly regulated to synthesize polypeptide chains. Nuclear export factor 1 (NXF1) binds to and transports the processed mRNA through the nuclear pore complex (Figure 4A) [32]. Free NXF1 forms a closed loop that hides its RNA binding domain and inhibits RNA binding. Therefore, coordinated binding of adaptors is required to transform NXF1 conformation to expose its RNA binding domain.

It has been reviewed that RNA-splicing is associated with nuclear export since the spliceosome can facilitate export factors binding to the mature RNA [33,34]. Canonical SR proteins exhibit different nucleo-cytoplasmic shuttling properties, grouped by shuttling to the cytoplasm (SRSF1, SRSF3, SRSF4, SRSF6, SRSF7, and SRSF10) or not (SRSF2, SRSF5, SRSF8, SRSF9, SRSF11, and SRSF12) (Figure 1) [11,35]. Therefore, SR proteins’ specific RNA-binding capacity and nucleo-cytoplasmic shuttling property endow them with functions in mRNA export. Recent work by Müller-McNicoll et al. analyzed transcriptomic-binding profiles of NXF1 and SRSF1–7 and suggested that, though all the examined SR proteins exhibit partial RNase A resistance in their interaction with NXF1, SRSF3 exhibits the most robust binding. Its motif is most similar to that of NXF1 in 3’ UTR, supporting the mechanism that SRSF3 serves as an adaptor in the recruitment of NXF1 to specific mRNA 3’ ends, coordinating RNA export [31].

### 4.2. Regulating mRNA Decay and Translation

Nonsense-mediated mRNA decay (NMD) has been considered a crucial strategy for protecting cells from the disastrous effects of truncated proteins or degraded transcripts containing premature termination codons (PTC) [36]. Interestingly, SR proteins can cross-interact with the exon junction complex (EJC)—the function of which is well-characterized in NMD, which suggests that SR proteins perform a significant role in NMD (Figure 4B) [37]. Additionally, several studies reported that overexpression of SRSF1, SRSF2, and other SR proteins, respectively, increase NMD, which is not observed with the overexpression of the splicing factor hnRNP A1 [38]. SRSF1 promotes NMD both after mRNA is exported to the cytoplasm and before mRNA is released from the nucleus [38]. Mechanistically, binding downstream of PTC, SRSF1 enhances the UPF1 recruiting to the spliced mRNA, which then promotes the NMD in the nucleus. Additionally, once the PTC is encountered, UPF1 is phosphorylated at its N and C termini, facilitating its binding with mRNA decay factors, e.g., SMG6 and SMG5/SMG7, and triggering RNA degradation. SRSF1 expedites UPF1 dephosphorylation by recruiting SMG7 and PP2A, facilitating UPF1’s releasing and recycling into the SURF complex [39].

In addition to the effect on NMD, SRSF1 is implicated in translational regulation. SRSF1 could increase the efficiency of eIF4E-initiated mRNA translation by facilitating the pioneer round of translation [40]. Another study revealed that SRSF1 co-sediments with the 80S fragment from the ribosome and polysomes, improving the translation of intronless reporter in HeLa cell-free translation system, which is dependent on the RS domain of SRSF1, but not on the RRM domain [41]. High-throughput sequencing of polysomal fractions further identified that mRNA translational targets of SRSF1 are associated with cell cycle regulation, including kinetochore formation, spindle assembly, and synthesis of M phase proteins, ensuring chromosome segregation (Figure 4C) [42]. Other shuttling SR proteins such as SRSF3 and SRSF7 can also function in the mRNA translation [43,44]. SRSF3, for instance, has been reported to interact with the internal ribosomal entry site (IRES), regulating poliovirus translation initiation (Figure 4C) [44]. In conclusion, SR proteins regulate gene expression by controlling multiple mechanisms throughout RNA processing.

## 5. Dysregulation of SR Proteins in Cancers

For their multiple functions involved in transcriptional and post-translational regulations, dysregulated SR proteins catastrophically disrupt genome stability, RNA metabolism, and transcriptome, thereby promoting tumorigenesis. Multiple lines of evidence suggest that SR proteins act as oncoproteins in different tumor types. SR proteins’ activities can be regulated by various mechanisms, including epigenetics, genomic mutation, expression level, and protein phosphorylation. Here we review how misregulated SR proteins facilitate tumor initiation and progression.

### 5.1. Aberrant SR Proteins Expression Is Associated with Cell Transformation and Tumorigenesis

The expression of SR proteins is generally dysregulated both in solid tumors such as colon, kidney, lung, liver, pancreas, and breast cancers [45,46,47,48,49] and in non-solid tumors such as leukemia and acute lymphoblastic leukemia (Table 1) [50,51,52]. As an oncoprotein, elevated SRSF1 expression has been reported in various cancers. Slight overexpression of SRSF1 can sufficiently transform fibroblast and mammary epithelial cells [47,53]. Won Cheol Park et al. pointed out an increased SRSF7 expression in gastric cancer in contrast to normal gastric mucosa [54]. Furthermore, colorectal cancer also exhibits increased SRSF3, SRSF5, and SRSF6 expression.

Several lines of evidence indicate frequent amplification of genes encoding SR proteins in different cancers, at least partially contributing to the high expression of SR proteins in tumors [45,47,69]. For example, extensive amplification of *SRSF6* has been identified in colon, lung, and breast cancers [69]. Meanwhile, *SRSF1* copy number gain is associated with DNA repair and chemo-sensitivity, which predicts poor survival in small cell lung cancers (SCLC) [45].

In addition to gene amplification, transcriptional regulations have also been reported to increase SR protein expression. MYC is a potent oncogenic transcription factor frequently hyper-activated in cancers [77]. Positive correlations between MYC and SRSF1 expression have been reported across different malignant contexts, including lung and breast cancers. Mechanistically, CHIP data show that MYC can bind to the SRSF1 promoter directly and enhance SRSF1 transcription [78]. Moreover, SRSF1 knockdown impaired MYC-induced cell transformation of Rat1a fibroblast, which in turn suggests the critical role of SRSF1 in MYC’s oncogenic functions [78]. Moreover, SR proteins expression can also be auto-regulated by individual SR proteins themselves or be cross-regulated by other SR protein family members via alternative splicing of ultra-conserved “poison exons” that are localized between protein-coding exons or in 3′UTR, and that contain a premature termination codon (PTC) associated with NMD-mediated mRNA degradation [9,79]. Further investigation of the auto- and cross-regulation mechanisms of SR proteins in cancer and how the loss of their regulation contributes to cancer should improve our understanding of the feedback loops of SR proteins expression and provide potential therapeutic opportunities.

Due to their canonical function in alternative splicing, the mechanism of dysregulated SR proteins promoting cancer progression has been well elucidated through aberrant regulation of alternative splicing and gene expression. For example, highly expressed SRSF1 in breast cancer promotes cancer progression via the oncogenic splicing switch of PTPMT1. In vivo CLIP assays identified a direct SRSF1 binding motif in PTPMT1 exon3 associated with exon inclusion. Expression of PTPMT1 isoforms respectively suggests that overexpression of exon3-included PTPMT1 isoform promotes, while exon3-excluded isoform suppresses tumor growth and metastasis [49]. Additionally, upregulated SRSF1, SRSF2, and SRSF3, respectively, are responsible for aberrant CD44 splicing in ovarian cancer [80]. Besides promoting tumor progression via aberrant regulation of alternative splicing, the function of highly expressed SR proteins has been implicated in aberrant translation in cancer. SRSF1 and SRSF9 have been suggested to facilitate β-catenin mRNA translation and promote β-catenin accumulation through mTOR signaling, which drives colon cancer progression [81].

### 5.2. Aberrant Phosphorylation of SR Protein in Cancer

Phosphorylation of protein can regulate their biological activity, subcellular localization, half-life, and docking with other proteins [82]. Subcellular localization of SR proteins is strictly regulated within mammalian cells via phosphorylation at multiple sites in the RS domain. Initially, phosphorylation in the RS domain could destabilize the α-helical structure, forming the “arginine claw” structures, which can be recognized by an SR-specific transportin (TRN-SR) that transports the splicing factors to the nucleus where the splicing occurs (Figure 4D) [83,84]. During spliceosomal assembling, the RS domain is appropriately phosphorylated [85]. Along with the splicing procedure, SR proteins are partially dephosphorylated during the late stage of splicing and then can be exported to the cytoplasm [86].

As the upstream regulator of SR proteins, the serine-arginine protein kinase (SRPK) can efficiently phosphorylate the RS domains of SR proteins and promote their nuclear import [87]. Recent studies pointed out that the phosphorylation of the RS domain is not random. SRPK1 phosphorylates the RS domain of its physiological substrate SRSF1 in a directional (C to N) mechanism. Pedro Serrano et al. revealed that, even though the RRM2 domain of SRSF1 is not the phosphorylation target of SRPK1, it coordinates the phosphorylation of the RS domain through cross-interaction between these domains. RRM2 interacts with the N-terminal RS domain to expose its C terminal for initiation of phosphorylation [88].

Aberrant SRPK1 expression can dysregulate SR proteins’ function, resulting in aberrant pre-mRNA splicing, which contributes to cancer progression [89]. SRPK1 contributes to lung and brain metastasis in patients with breast cancer, which is associated with prognosis [90]. Inhibition of SRPK1 excludes SRSF1 from the nucleus and affects alternative splicing of VEGF to inhibit angiopoiesis, suppressing the prostate cancer cell line PC-3 forming tumors in vivo [91].

Dysregulated expression of another SR protein kinase, Cdc2-like kinases 1 (CLK1), has been reported in pancreatic ductal adenocarcinoma, which enhances phosphorylation on SRSF5^250-Ser^ that is associated with alternative splicing of METTL14 and Cyclin L2. Aberrant METTL14 exon 10 exclusion enhances the N6-methyladenosine modification in PDAC cells which promotes tumor metastasis, while aberrant splicing of Cyclin L2 exon 6.3 promotes tumor proliferation [67].

Protein kinase A (PKA), a novel tumor biomarker, phosphorylates SRSF1 at serine 119 in the RRM, which enhances the RNA-binding properties of SRSF1, and increases SRSF1′s activity in regulating the Minx transcript splicing in vitro [92]. Hyperactive PKA signaling pathway has been reported in different cancers and can be exploited as potential cancer therapeutic and diagnosis [93].

Hypoxia is commonly recognized as a prognostic factor negatively related to therapeutic response and cancer patients’ survival [94]. Increasing hypoxia-inducible factor HIF-1 in hypoxic cells upregulates the transcription level of CDC-like kinase 1 (CLK1), which subsequently generates hyperphosphorylated RS domain of SR proteins and decreases their RNA-binding specificity. Consequently, infrequent or new isoforms were generated in hypoxic tumors compared with the normoxic environment, contributing to tumor progression and resistance to radiation therapy.

Overall, SR proteins can be phosphorylated at multisite by different kinases, which comprehensively adjust SR proteins’ activity. Further investigating the underlying mechanisms of the spatiotemporal phosphorylation of SR proteins associated with subcellular distribution and protein–protein interaction should improve our understanding of SR proteins’ role in tumorigenesis and progression.

### 5.3. SR proteins Link Alternative Splicing and Epigenetics Promoting Cancer Development

Aberrant epigenetic regulation, a susceptible adapter of multiple biological changes, increases the risk of cancer [95]. SR proteins have been implicated as potent mediators for aberrant epigenetic-mediated tumorigenesis and metastasis. For example, histone methylation affects the affinity of SR proteins to their specific RNA binding sites. DNA methylation allows for the H3K9me3 modification of histone corresponding to methylated alternative exons. As a histone methylation reader, HP1 protein recruits SR proteins to the transcribed nascent RNA, thus leading to specific alternative splicing outcomes [96]. Then SRSF1 and SRSF3 bind directly to HP1α and HP1 via RNA-independent interaction. However, in the absence of HP1, overexpression of SRSF3 resulted in a diminished effect on splicing and vice versa. These results indicate that SR proteins provide bridging between alternative splicing and epigenetics. Further research dissecting the underlying mechanism of the SR proteins as executors of epigenetic changes should improve our understanding of SR proteins’ roles in physiological conditions and tumor development.

## 6. Competition or Coordination between Different SR Proteins

**Figure 5:**
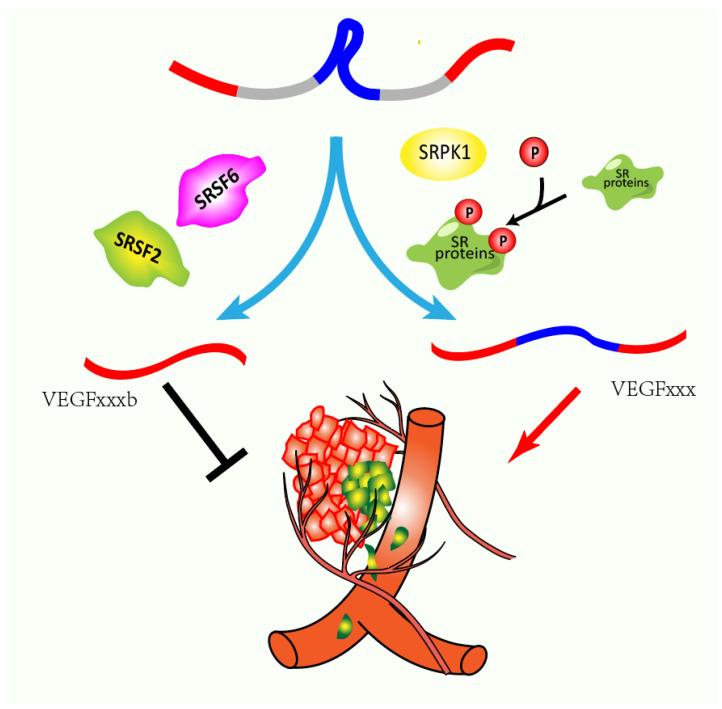
Co-regulation of SR proteins on common target through multiple mechanisms. As a contributor to angiogenesis, dysregulation of VEGF splicing contributes to cancer progression and is highly correlated with acquired drug resistance. For example, upregulated expression of SRSF2 or SRSF6, as a result of transcription increased or copy number amplified, is responsible for splicing in favor of VEGF165b, which is antagonistic to tumor neovascularization in vivo. However, overactivity of SRPK1 in the tumor could phosphorylate SRSF1 and stimulate its nuclear import, which favors the selection of the proximal splice site of VEGF pre-mRNA and promotes neovascularization.

Specific splicing target is often regulated synergically by multiple splicing factor proteins [97]. As a typical target for SR proteins, alternative splicing of VEGF mRNA leads to different isoforms with diverse functions. Particularly, the VEGFxxx isoforms and the VEGFxxxb isoforms (x denotes the number of amino acids) show opposite roles with pro- or anti-angiogenic properties, respectively (Figure 5) [98,99]. It has been reported that dysregulated VEGF splicing is associated with cancer progression. Decreased VEGFxxxb has been reported in several cancers, including renal [100], melanoma [101], and colorectal carcinoma [102]. Different SR proteins regulate VEGF splicing via multiple mechanisms. Aberrant expressions of multiple SR proteins disrupt the VEGF splicing. For example, it has been found that increased expression of SRSF2 can upregulate the VEGF165b/VEGF ratio and suppress tumor neovascularization [103]. Similarly, SRSF6 can bind directly to the exon 8b of VEGF pre-mRNA favoring distal splice site utilization and VEGF_165_b expression [104]. On the contrary, increasing SRPK1 in tumor phosphorylates SRSF1 and stimulates its nuclear import, which favors the splicing at the proximal splice site of VEGF pre-mRNA and leads to the expression of the VEGFxxx isoform and neovascularization. The mechanism for the competition or coordination between different SR proteins during individual splicing events needs to be further explored to clarify the exact roles of SR proteins and their cross-interaction in splicing. Moreover, investigating other cross-interactions between SR proteins during RNA processing, mRNA export, mRNA stability, and translation will expand our understanding of their functions in physiological and pathological conditions.

## 7. Targeting SR Proteins as Potential Cancer Therapeutics

To target SR-proteins-mediated aberrant alternative splicing in cancer, a vaccine approach has recently been developed to apply antibodies specifically to VEGFxxx, and not to VEGFxxxb isoforms, providing a promising strategy for cancer therapy. A prophylactic immunization using purified immunoglobulins against the pro-angiogenic isoform of VEGF suppresses melanoma and renal cell carcinoma tumor growth in vivo [105].

**Figure 6:**
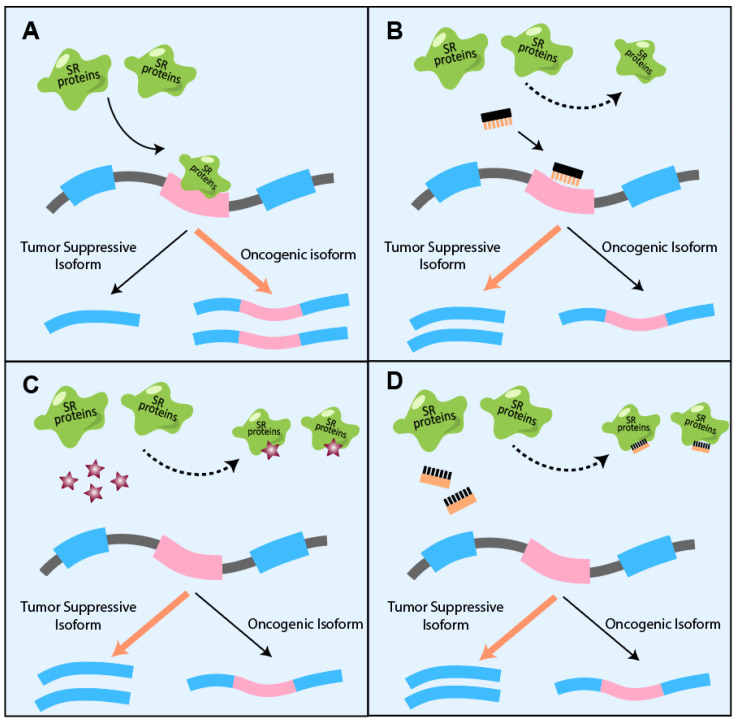
Strategies for correcting SR-protein-mediated aberrant alternative splicing. (**A**) Elevated SR proteins alter RNA splicing. (**B**–**D**) Principles for targeting SR proteins induced aberrant RNA splicing in cancer. (**B**) Antisense oligonucleotides bind to RNA and block SR proteins’ effect on target RNA alternative splicing. (**C**) Small molecular inhibitors bind to RRM domain of SR proteins and inhibit RNA binding. Stars represent small molecular inhibitors. (**D**) Decoy oligonucleotides composed of SR protein binding sequence bind to SR proteins and block their access to the target RNA.

Importantly, with advances in the development of antisense oligonucleotides (ASOs)—synthetic molecules with 15–30 nt in length, base-pair binding to the splice sites or regulatory sequences [106]—have been under pre-clinical evaluation for correcting aberrant alternative splicing in cancer (Figure 6A,B). SRSF3 recognizes and enhances the inclusion of the exon 10 of the pyruvate kinase M (PKM) that plays a crucial role in regulating the Warburg effect, favoring the M2 isoform that promotes aerobic glycolysis and tumor growth [107,108]. Another study suggested that highly expressed SRSF10 in head and neck cancer is also associated with aberrant PKM splicing resulting in the expression of PKM2 isoform [109]. ASO targeting PKM splicing increases the PKM1 to PKM2 ratio, thereby redirecting glucose carbons from anabolic processes to the tricarboxylic acid (TCA) cycle in hepatocellular carcinoma and suppressing the liver-tumor formation and growth [110].

In addition to RNA alternative splicing, dysregulated SR proteins can disrupt genome stability, transcription, and further RNA processing, contributing to tumorigenesis and progression. Therefore, targeting SR proteins instead of their splicing targets may modulate gene expression extensively and benefit tumor therapeutics. In order to modulate SR protein phosphorylation, small molecules have been developed to target the kinases associated with splicing regulation [111,112,113]. NB-506, a complete inhibitor of topoisomerase I, is repositioned to reduce SRSF1 phosphorylation and regulates gene expression by modulating pre-mRNA splicing. In addition, it has been described that chimeric antibodies of SRPK1 have the capacity to suppress non-small cell lung cancer growth and metastasis [114]. Meanwhile, small molecules targeting SRPK1 can switch splicing of VEGF mRNA to generate the VEGF165b isoform and decrease tumor growth [91]. These studies suggest that inhibition of SRPK1 may provide a strategy for cancer therapeutic.

Modulating the SR protein kinases’ activities tends to change multiple SR proteins’ functions simultaneously. Several efforts have been made to specifically target the individual hyperactive SR proteins in tumors (Figure 6C,D). Virtual screening of 4855 FDA-approved drugs to target the domain-specific role of SRSF6 repurposed a β2-adrenergic receptor agonist indacaterol, which is approved for chronic obstructive pulmonary disease (COPD) treatment, for blocking SRSF6′s regulation on its downstream splicing targets, which suppresses colorectal cancer [48]. In addition, Denichenko et al. developed decoy oligonucleotides that can compete for RNA binding proteins, such as splicing factors, including RBFOX1/2, SRSF1, and PTBP1, rather than their specific RNA targets. SRSF1 splicing targets, such as INSR, U2AF1, MKNK2, and USP8, respond to decoy oligonucleotides for SRSF1 while not to those for PTBP1 and RBFOX1/2. Considering the non-RNA-binding functions of SR proteins, strategies targeting the splicing regulation activity of SR proteins may provide more precise on-target therapeutic benefits.

## 8. Conclusions

In summary, SR proteins’ activities are regulated via multiple mechanisms, including gene copy number [47], transcriptional regulation [50], alternative splicing [9,79], and phosphorylation [91], which affects a variety of cellular processes, such as genome stability [12,115], RNA metabolism [116], and translation [117]. Connecting upstream regulators and downstream targets, multiple SR proteins shuttle between the nucleus and cytoplasm to maintain the precise net of the biological pathways. Dysregulated SR proteins drive tumorigenesis and cancer development [45,46,47]. To date, a few therapeutic strategies have been developed to regulate the activity of SR proteins [48,111,112,113,118]. Among them, targeting the regulators of SR proteins will result in extensive modulation of various SR protein functions simultaneously, which may elicit unwanted off-target effects. On the other hand, ASOs targeting specific aberrant alternative splicing changes may confer limited clinical benefit in cancer therapy, considering that hundreds and thousands of transcripts are aberrantly spliced in cancer due to the dysregulated SR splicing proteins. Therefore, we speculate that the protein-specific competitive inhibitor or decoy oligonucleotides targeting individual SR proteins can achieve more significant benefits for cancer therapeutics.

Further investigation of the biological function and regulatory mechanisms of SR proteins in RNA metabolism and distinguishing the cross-interaction among different high-homology SR proteins should provide a foundation for the rational development of precise cancer therapy (Table 1). It has been reported that SR proteins such as SRSF1, SRSF4, SRSF5, SRSF6, and SRSF9 contain one more RRM domain than the other family members [119]. Further insights into the functional discrimination of two RRM domains are needed to specify their respective roles in RNA recognition and other RNA processing. With further understanding of the biological function of the protein domains in SR proteins, SR proteins may serve as a precise medicinal target for cancer therapy.

## Figures and Tables

**Table 1:** The effects of SR proteins in cancer cells.

Splicing Factor Name	Cancer Type	Changes of SR Proteins in Cancer	SR Proteins’ Effect
SRSF1	Lung Cancer	Protein [55,56] Phosphorylation [57]	Radioresistance [55] Autophagy [56] Apoptosis [57]
	Breast Cancer	Protein [53] mRNA [49]	Apoptosis [49,53] Cell cycle arrest [49]
	Colon Cancer	mRNA [58]	DNA Damage [58]
	Glioma	mRNA and Protein [59]	Cytoskeleton reorganization [59]
	Renal Cancer	mRNA [60]	Apoptosis [60]
SRSF2	Lung Cancer	mRNA [61]	Angiopoiesis [61]
	Liver Cancer	mRNA and Protein [62]	Proliferation [62]
	Renal Cancer	mRNA [60]	Apoptosis [60]
SRSF3	Ovarian Cancer	mRNA [63]	Apoptosis [63]
	Colon Cancer	Protein [64]	Angiogenesis [64]
	Oral Cancer	Protein [65]	Autophagy [65]
	Glioma	mRNA [66] Protein [66]	Cell Mitosis [66]
SRSF4	Acute myeloid leukemia	mRNA [50]	Apoptosis [50]
SRSF5	Pancreatic Cancer Breast Cancer	Phosphorylation [67] Protein [68]	Cell Cycle [67] Apoptosis [68]
SRSF6	Colon Cancer Lung Cancer	mRNA [69] Protein [48]	Tumorigenesis [69] Apoptosis [70] Cell–cell junction [48]
SRSF7	Colon Cancer Lung Cancer	Protein [71] mRNA [72]	Apoptosis [71] Growth Arrest [72]
SRSF9	Colon Cancer	mRNA [73] Protein [73]	Ferroptosis [74] m6A Modification [73]
SRSF10	Cervical Cancer	mRNA [75,76] Protein [75]	Macrophage Phagocytosis [75] Nonsense-mediated mRNA decay [76]

## Data Availability

Not applicable.
